# Children’s Perceived Barriers to a Healthy Diet: The Influence of Child and Community-Related Factors

**DOI:** 10.3390/ijerph19042069

**Published:** 2022-02-12

**Authors:** Paula Magalhães, Catarina Vilas, Beatriz Pereira, Cátia Silva, Hélder Oliveira, Camila Aguiar, Pedro Rosário

**Affiliations:** Department of Applied Psychology, School of Psychology, University of Minho, 4710-057 Braga, Portugal; catvovilas@gmail.com (C.V.); beatriznpereira94@gmail.com (B.P.); catiasbsilva@gmail.com (C.S.); a81780@alunos.uminho.pt (H.O.); camiladeaguiar@gmail.com (C.A.); prosario@psi.uminho.pt (P.R.)

**Keywords:** perceived barriers, children, content analysis, healthy diet, Six C’s Model

## Abstract

A healthy diet influences the promotion and maintenance of health throughout an individual’s life. Many individuals struggle to have a healthy diet, despite it being mainly under their control. The current study aims to explore children’s perceived barriers to a healthy diet. A qualitative study with the open-ended question, “Please identify the top 5 barriers to a healthy diet”, was undertaken between January–June 2019 in which 274 students from the 5–6th grades wrote down their answers to the open-ended question. Content analysis was used to analyze responses with a codebook based on the Six C’s Model. Five categories were identified: Child, Clan, Community, Country, and Culture-related barriers. Findings showed that the barriers most highlighted were in the Child sphere (e.g., dietary intake) and the Community sphere (e.g., peer food choices). Children seldom referred to barriers from the Clan sphere, i.e., related to family (e.g., food available at home). Additionally, it seems that girls emphasize more barriers from the Child sphere, while boys emphasize more barriers from the Community sphere. Due to the qualitative nature of this study, interpretation of the data should take into account the specific characteristics and context of the sample. Nevertheless, the current data are helpful in identifying implications for practice, for example, the need to empower children with tools (e.g., self-regulation-based interventions) likely to help them overcome perceived barriers. Finally, advocacy groups may help set environmental and structural changes in the community likely to facilitate children’s healthy choices.

## 1. Introduction

A healthy diet is an important factor greatly influencing the promotion and maintenance of health (e.g., preventing chronic diseases, obesity). However, many individuals struggle to initiate and/or maintain a healthy diet, despite it being mainly under their control. [1,2,3,4]. For example, while the World Health Organization (WHO) recommends that every child eat five servings of fruit and vegetables per day while limiting salt, sugar, and fat intake [5], actual food consumption patterns still fall short of the recommendations. Literature and international reports show that children still consume large amounts of energy-dense foods and foods high in fat, sugar, and salt, while consuming low amounts of fiber, vegetables, fruits, and whole grains [5,6,7]. Previous research has analyzed factors associated with adopting a healthy diet to better understand the reasons likely to inhibit healthy eating habits.

**Figure 1:**
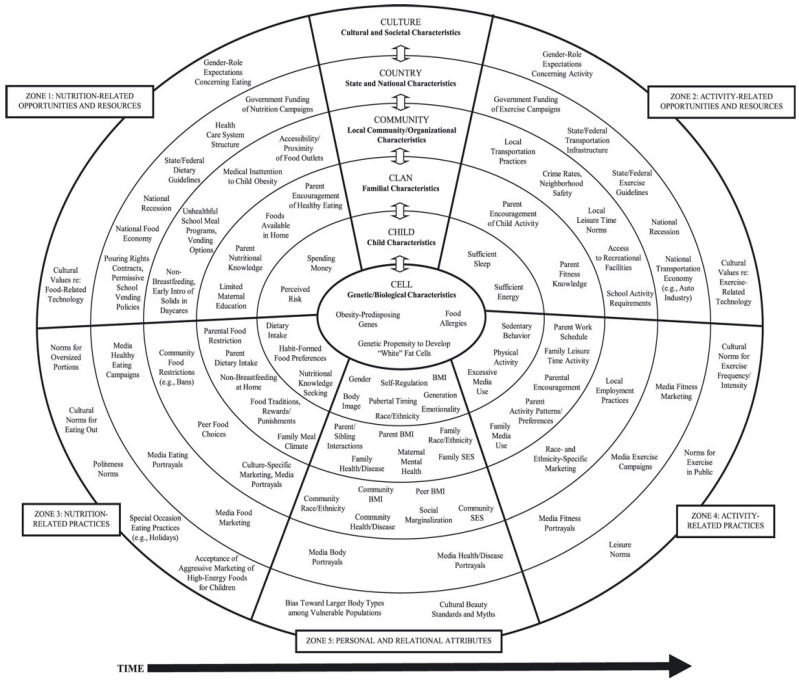
The Six-Cs developmental ecological model of contributors to overweightness and obesity in childhood. The model was reproduced with the permission of the first author, Kristen Harrison [12].

While examining predictors of children’s eating habits, researchers have explored individual-related factors (e.g., knowledge-related) and external factors (including social, economic, and cultural) influencing a healthy diet [8,9,10,11]. The current study aimed to further analyze these factors on children’s eating habits, with the Six C’s model providing a relevant theoretical framework to understand these factors. The Six C’s Model [12] is a developmental, ecological model that summarizes a vast body of research focused on factors likely to influence childhood obesity, including factors related to eating behavior (Figure 1). The model is organized into six spheres, ranging from internal to external influences, namely: (1) Cell: this sphere represents genetic/hereditary and biological factors. For example, food allergies that limit the type of foods children include in their diet [13]. (2) Child: this sphere represents children’s personal and behavioral factors, most of which are under their control. For example, excessive media use (e.g., watching television) is commonly associated with a daily increase in energy intake [14,15]. (3) Clan: this sphere represents family factors, for example, eating behaviors observed at home [16]. (4) Community: this sphere represents all factors pertaining to social contexts (e.g., peers, school). For example, accessibility or proximity of food outlets is associated with an increased caloric intake [17]. (5) Country: this sphere represents national characteristics, for example, countries in which advertisements showcase calorically high and nutrient-deficient foods, which may influence children’s preferences [18]. (6) Culture: this sphere represents cultural and societal characteristics that influence people’s beliefs and behaviors regarding eating and food choices. For example, cultural beauty ideals are likely to shape standards towards body image and, consequently, influence individuals’ choices/opinions [19].

As previously mentioned, research has been highlighting several important factors influencing children’s healthy eating (e.g., nutritional knowledge, and social, economic, cultural, and psychological factors) and this corpus of research has been informing the design of subsequent interventions [12,20]. However, the barriers to a healthy diet from the children’s point of view have been receiving little attention from researchers. This is relevant as the literature suggests that the match between participants’ needs and the intervention’s content and goals is crucial for overall engagement with the intervention and, consequently, the adoption of a healthy diet [21,22]. Acknowledging this gap in the literature, the main goal of this study is to deepen the understanding of children’s perceived barriers to adopting or maintaining a healthy diet. Findings are expected to provide data likely to help interventions match children’s needs and increase the efficacy of interventions focused on promoting healthy eating. Moreover, this study aims to comprehend the role that children’s sex and family income may play in their perceived barriers. This particular goal was included for two reasons. First, there is evidence that boys follow unhealthier diets than girls [23,24,25]. Second, data indicates that children from low socioeconomic backgrounds make poorer dietary choices than their non-low socioeconomic background counterparts [4,26]. This may be due to these families having fewer resources and opportunities to buy healthy foods (e.g., a healthy diet is about $1.50/day more expensive than a less healthy one) and incorporate healthy dietary patterns into their lives [4,27,28,29].

## 2. Materials and Methods

### 2.1. Context and Procedure

This study is part of a large research project approved by the University of Minho’s Ethics Committee for Research in Social and Human Sciences (CEICSH) (CEICSH 032/2019). The research project aimed to develop a hybrid healthy eating promotion intervention for elementary school children, consisting of face-to-face sessions that were complemented by a concurrent online component. This intervention took place in two schools in the north of Portugal between January and June 2019 (to learn more about the intervention, see Magalhães et al., 2020) [30]. The intervention comprised 18 sessions, in which the first half was devised to promote self-regulation strategies (e.g., how to set goals; how to plan, execute, and evaluate the behavior to attain the goal), and the second half was devised to transfer knowledge of self-regulation strategies to the healthy eating domain. Prior to the transference sessions, a session about children’s perceptions of the obstacles of healthy eating was carried out. The present study derives from this session, in which students were presented with a prompt describing the global rates of obesity and invited to answer a question about the barriers to a healthy diet (see the materials and measures section). Children took approximately 15 min to complete the task.

Consent to conduct the study in a school setting was obtained from the Portuguese Ministry of Education, and written informed consent and assent was obtained from parents/caregivers and children, respectively, from this convenience sample. Codes were assigned to participants to protect data confidentiality and anonymity.

### 2.2. Participants

The present study consisted of 274 children from the 5th and 6th grades, ranging from nine to 14 years old (M = 10.9, SD = 0.86, Mdn = 11), of which 52.2% were boys. Of the 274 participants, 100 (36%) were from low-income families. Please note that the characteristics of the participants may not be representative of the population.

### 2.3. Materials and Measurements

#### 2.3.1. Sociodemographic Questionnaire

Participants’ demographic information included age, sex, and year in schooling.

#### 2.3.2. Family Income

Family income is the best predictor of food purchasing behavior [28]. Participants’ families’ income information was obtained through the School Social Welfare office. The families of students applying for school welfare assistance (e.g., free lunch, a voucher to buy school materials, textbooks) are grouped according to their income. Level A corresponds to a family income of up to 3050.32 euros per year ($3544.46), Level B up to 6100.64 euros per year ($7088.91), and Level C up to 9150.96 euros per year ($10,633.37) [31]. Families with higher incomes are not eligible for this assistance program. It is worth noting that, in Portugal, the minimum wage in 2019 was 600 euros per month ($697.20), i.e., 8890.0 euros per year ($10,330.14) (DGERT/MTSSS, PORDATA, 2021). The present study classified family income as echelon A, B, and C for low income and no echelon for median/high income.

#### 2.3.3. Barriers to Healthy Eating

During the data collection session, the research assistant showed a scenario (see below) describing the global rates of obesity along with a statement asserting that unhealthy food consumption is an important factor that may contribute to this situation. Children were then asked to identify and elaborate on the “Top 5 Barriers to a Healthy Diet”.

“It is estimated that: >1.1 billion adults have overweightness, and 312 million have obesity; 10% of children have overweightness or obesity, and 17.6 million under the age of 5 have overweightness; Please, write down the TOP 5 barriers to a healthy diet:”

Due to the explorative nature of the current study, an open-ended question was chosen as the data collection tool. This methodological option prevents the likelihood of biased responses and is better suited to capture participants’ diversity of conceptions about a topic, compared to Likert scales [32,33].

### 2.4. Data Analysis

**Figure 2:**
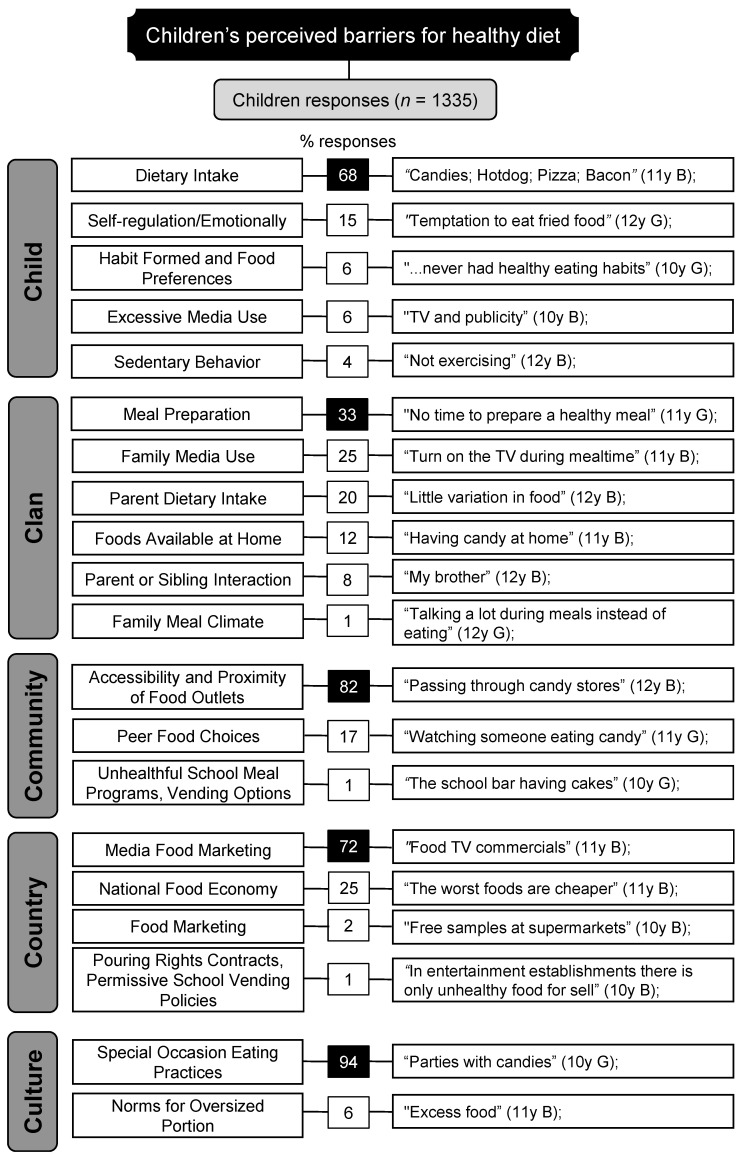
Categories, subcategories, response frequency, and representative quotes.

To summarize demographic data, descriptive statistical analyses were conducted using the software IBM SPSS Statistics, version 26.0. Qualitative data were organized and coded using the software NVivo 10 (QSR International version 10). This study followed the steps of content analysis by Bardin [34] (1) pre-analysis, (2) exploration of the data, and (3) treatment. In the first step, researchers read all participant responses to become familiar with the data. While exploring the data, researchers followed a deductive and inductive approach to identify the categories and subcategories. First, all categories and subcategories were established a priori in a codebook based on the spheres and factors within each sphere of the Six C’s Model [12] (Figure 2). Nonetheless, a new subcategory emerged from the data and was added using the general idea of the participants’ answers (e.g., “meal preparation”). Consequently, all material previously coded was reviewed and checked against this new subcategory. Two researchers coded 100% of the data independently. All differences were resolved through discussion to reach a consensus, resulting in an inter-rater reliability of 0.86. Finally, in the last step of content analysis, researchers checked the number of responses and respective percentages for each category and subcategory. Finally, a matrix-coding query was conducted considering the participants’ attributes: sex and family income.

## 3. Results

The current study explored the barriers to a healthy diet as perceived by Portuguese children in the 5th and 6th grades. In total, 1289 responses were collected, 11 of which were excluded for not being valid (e.g., “my cat” CFMS7, 12 year old boy). Finally, 1278 responses from 274 participants were analyzed. Of the 274 participants, 227 indicated five barriers, 17 indicated four barriers, 18 indicated three barriers, nine indicated two barriers, and three indicated one barrier. Note that several responses were coded in more than one subcategory; therefore, the total sum of the subcategory’s response frequencies is 1335. For example, the answer “Laziness towards cooking” (DJRS5, 11-year-old boy) was coded simultaneously within the “self-regulation/emotionality” and “meal preparation” subcategories.

Five major categories were identified: (1) Child, (2) Clan, (3) Community, (4) Country, and (5) Culture. Figure 2 presents the subcategories that emerged in each category with representative quotes and their response frequency. Note that, except for the subcategory “Meal Preparation” all categories and subcategories match the spheres and factors comprising the Six C’s Model [12], respectively. A detailed description of the main findings is presented below.

### 3.1. Child

When participants indicated barriers within the child’s ability to change, those responses were classified in the Child category, the most frequent category, making up 71% of responses. Specifically, the most common barrier (subcategory) as described by children to a healthy diet was Dietary Intake. For example, participants referred to specific foods or food components, mainly calorically dense foods, as distracting to their eating behaviors: “Juice, chocolates, lollipops, cakes, and chips” (GNC10, 10y G) and “Drinks with too much sugar” (A36, 10y G). This may indicate that children lack self-regulation skills to help them cope with unhealthy foods and make healthier choices. In fact, participants expressed difficulties controlling their food cravings triggered by smelling or seeing palatable food. Examples of responses coded in the subcategory Self-Regulation/Emotionality include “Temptation to eat delicious candies” (AF4, 13y B) and “Seeing a sweet food and feeling like eating it” (AF1, 10y B). Still, participants also indicated the lack of motivation to follow a healthy diet as a barrier: “Not wanting to eat healthy foods” (HCR12, 11y B) and “There is little motivation for a healthy lifestyle” (ABBC4, 11y G).

Another subcategory identified in the Child sphere is Habit-Formed or Food Preference. Children reported that liking unhealthy foods or disliking healthy foods and having unhealthy daily food habits or traditions constitutes a barrier to following a healthy diet: “Not liking vegetables” (IGG11, 11y G) and “We want to go on a [healthy] diet, but we don’t succeed because we are used to junk [food]” (MPR21, 10y G). Additionally, participants mentioned Excessive Media Use as a relevant barrier to practicing healthy eating. Children’s responses suggest that excessive screen time could influence their food choices. For example, “Social media showing processed foods” (FC8, 11y G). Lastly, Sedentary Behavior was another barrier to a healthy diet mentioned by the participants, “Spending a lot of time on the computer” and “Not doing physical exercise” (CGA4, 12y B).

### 3.2. Clan

Participants’ responses were included in the Clan category whenever they mentioned barriers related to family characteristics. Curiously, only 6% of the responses referred to Clan-related barriers. Within this category, the major perceived family-related barrier was Meal Preparation. Children indicated that parents/caregivers lack time and energy to prepare healthy and balanced meals. Therefore, families often choose a fast and easy meal, mainly composed of unhealthy types of food, such as fast food: “For instance, it is easier to cook sausages than to cut some vegetables and wash them” (ALVB6, 11y G) and “Running out of time and choosing fast food” (LA15, 11y B). Children also indicated that Family Media Use, such as using digital devices during family mealtime, namely watching TV, could be a barrier to a healthy diet. Examples include “Eating and watching TV [at the same time], and then we find out that the food is still on the plate” (AR2, 10y G) and “Playing with the smartphone while eating” (IFS12, 11y G). Participants also highlighted Parent Dietary Intake as an important factor influencing their eating patterns: “Parents instill in their children habits to eat poorly from a young age” (ARM6, 11y G) and “Go too many times to restaurants” (TMMCF30, 11y B). Participants also identified Foods Available at Home as another barrier. Usually, parents/caregivers are responsible for deciding what type of food is bought and available at home. For example, parents/caregivers may choose to buy calorically dense foods: “Having candy at home” (MST19, 11y G) and “[having] Too many cookies in the pantry” (TMMCF30, 11y B).

### 3.3. Community

Community-related barriers, i.e., barriers related to the social world outside the home, was the second most frequent category (13%). Children identified community constraints as barriers to a healthy diet, with the subcategory Accessibility and Proximity of Food Outlets being the most reported. Some participants’ answers suggested that they experienced difficulties controlling the impulse to eat unhealthy foods when food stores or restaurants, mainly fast-food restaurants, were easily accessible: “When I pass through McDonald’s, I want to eat right away” (DBM2, 11y B) and “Having too many cakes available in the place we are going is difficult…” (MCC22, 11y G). Children also mentioned Peer Food Choices as a barrier to healthy eating. They indicated that peers and friends could influence their food choices in many ways. Participants mentioned struggling with several situations: (1) Refusing an invitation from a friend to go to a fast-food restaurant (“Friends’ invitation to go to McDonald’s” (FPFG8, 11y G); (2) Watching a friend eating unhealthy food while controlling the urge to eat the same food item (“Seeing my classmates buying things at the (school) bar/cafeteria” (LV17, 11y G), “Being in a restaurant eating vegetables and watching someone eating meat with French fries” (FSA7, 12y B)); (3) Refusing a friend’s offer of an unhealthy type of food [“Offering me sweets or unhealthy food” (MFVRM24, 11y G)]. Both Accessibility and Proximity of Food Outlets and Peer Food Choices seem to reveal that participants lack self-regulation strategies to cope with external barriers and influences.

### 3.4. Country

Country or national constraints were also mentioned as playing an important role in food options, representing 8% of responses. Media Food Marketing was the main barrier to a healthy diet reported by participants. Children indicated that publicity, mainly food commercials, was likely to encourage individuals to buy and eat the food advertised: “Advertisement leads people to consume unhealthy products” (MJTC22, 11y G) and “Fast food ads” (RCCV25, 11y B). Furthermore, National Food Economy was the other emerging subcategory within the Country category. Children reported that national food prices influence food options. For example, participants mentioned that unhealthy food is cheaper than healthy food, and that the difference in prices is an important barrier to eating healthily: “Fast food is (…) cheaper” and “Healthy products are more expensive” (ABBC4, 11y G). Participants also expressed that discounts and promotions work as triggers to buy unhealthy foods: “Discounts on food that is bad for us” (MAC19, 12y G) and “Go to a supermarket with cookies on sale” (TMMCF30, 11y B).

### 3.5. Culture

Cultural norms related to food consumption was the least prominent category (2%). Nonetheless, children identified Special Occasions Eating Practices as a barrier to a healthy diet, i.e., children mentioned that local culture influences eating patterns, particularly in special moments or specific contexts, due to implicit food “rules” that encourage individuals to eat certain foods. For example, there is a broad consensus on the association between watching a movie in a cinema and eating popcorn. The cinemas have sweet or salty popcorn and a vast array of soda options for sale. Thus, when children go to the cinema, they expect to eat popcorn, not other types of food. Children added other examples: “Candies and sodas at birthday parties” (MCC20, 11y G), “At Easter, they give us chocolate eggs” (ROP27, 11y B).

### 3.6. Child Sex and Family Income Differences

We found similar results in responses for nearly all categories when considering sex and family income. However, data suggests that girls and boys emphasize different barriers to healthy eating. Specifically, girls mentioned 7% more Child Sphere type of responses compared to boys. Moreover, boys mentioned 9% more Community Sphere type of responses compared to girls. In addition, the frequency of categories or subcategories identified did not differ between children from low-income backgrounds and those from median or high-income backgrounds.

Table 1 systematizes information regarding the distribution of the responses per category, with the influence of sex and family income in consideration. Please note that percentages were not calculated by category, i.e., spheres, but by the attributes, i.e., sex and family income. Otherwise, the percentages would be proportional to the number of participants in each attribute.

## 4. Discussion

Following a healthy diet is a protective behavior against several health problems [1,3,4]. However, children’s diets still fall short of the recommended international guidelines [6,7]. The present study focused on understanding children’s perceived barriers to a healthy diet, as we believe that gathering data on this topic is crucial in helping families directly and is valuable in designing school-based programs on the topic. The Six C’s Model [12] guided the data analysis. Findings point to a complex set of barriers preventing or hindering children from making healthy food choices (Figure 2). The most frequent category, Child-related barriers, included factors proximal to children and perceived as under their control and/or direct influence (e.g., Dietary Intake). The other barriers described factors that are distal and uncontrollable; for example, children’s reports greatly emphasize Community-related barriers (e.g., Accessibility and Proximity of Food Outlets).

Data in the Child category included nutrition-related and sedentary activity-related barriers that deeply influence children’s dietary intake. For example, regarding nutrition-related barriers, participants expressed difficulty controlling their urges to eat specific foods, mainly unhealthy foods, due to smelling or seeing them while walking in the street. In addition, children mentioned that the habit of eating palatable food (e.g., fast food) makes it difficult to include healthier and less salty foods in their diet. These findings are consistent with those of Pereira et al. [35], suggesting that children may lack the self-efficacy beliefs and self-regulatory skills needed to help them focus on the goal of following a healthy diet. Regarding sedentary behavior, participants stated that engaging in sedentary activities, such as the intense use of screen time, could work as a barrier to healthy eating in two different ways: (1) as a distraction that leads to a decreased intake of food. In fact, not finishing a healthy meal could be an unhealthy behavior. (2) As an encouragement to consume unhealthy foods. This second point is consistent with previous research indicating that engaging in certain activities, such as watching TV and using smartphones, induces children to stay at home and consume high-calorie foods in excess [15,36].

Concerning the Community category, data indicate that children’s social environment could function as a barrier to healthy eating. For example, easy access to neighborhood convenience stores and fast-food restaurants was reported as a barrier to adopting healthy eating habits. Previous studies reported that having fast food restaurants surrounding homes and schools encouraged fast-food consumption [8,9]. In the present study, participants reported feeling tempted when they see, smell, or pass through those places. In addition, participants expressed difficulties avoiding restaurants to which their friends and classmates go or invite them. This finding suggests that factors outside the home environment are likely to influence children’s eating patterns, such as their peers and social networks. Beck and colleagues’ [8] work, focused on peer influences, reported similar findings. These authors found that peers’ influence on children and adolescents’ eating patterns may be either negative or positive towards their daily consumption of calorically dense foods or healthy foods, respectively.

Children seldom refer to Clan-related barriers, i.e., parent and family characteristics and behaviors. This finding was unexpected because prior literature has stressed the active role of parents and family in the establishment and promotion of children’s eating behaviors [36,37,38,39]. It is possible that due to their age, children may lack the maturity to understand and show difficulties in emotionally distancing themselves. These limitations may prevent them from perceiving their family’s routines as potential barriers to healthy eating [40]. Thus, there may be a metacognitive limitation in conceptualizing family routines as barriers to healthy habits, such as watching TV while eating or ordering take-out food. Therefore, future research may consider exploring the possibility that children do not perceive the family’s established routines as barriers to healthy eating through semi-structured interviews. Interestingly, the lack of self-regulation to cope with healthy eating barriers seems transversal to most categories. For example, in the Clan category, parents who lack time and energy to cook due to their busy lives are likely to opt for a fast, easy, and unhealthy meal (e.g., fast food). Furthermore, in the Community category, children admitted struggling to resist temptations such as friends offering unhealthy foods. Overall, these findings are consistent with the previous literature warning that motivational-related factors are key to adopting a healthy diet [41,42,43]. Thus, training children with self-regulation strategies (e.g., setting goals for healthy eating (e.g., when my stomach “grumbles”, I choose to eat one piece of fruit), putting crisps out of reach as a strategy to avoid them when watching television) may be a relevant tool to help children face the perceived barriers to a healthy diet [43,44].

Finally, girls mentioned barriers in the Child category more often than boys, and boys mentioned barriers in the Community category more often than girls. These results may help explain why boys are more prone to following unhealthy diets than girls [23,24,25]. In fact, while reporting barriers from the Community category, boys highlighted the external nature of those barriers and the impossibility of removing them, as they are not under their control. Existing research stresses that explaining an outcome through external factors not under the control of the individual contributes to feelings of inefficiency and the passive acceptance of circumstances and hardships [45]. Thus, individuals who rely on external factors to explain outcomes are likely to feel hopeless in the face of uncontrollable non-behavioral factors [45]. Therefore, consistent with the literature, boys believing that they do not control their food intake are not expected to make efforts to adopt healthy eating habits [35]. Conversely, girls who mentioned barriers in the Child category highlighted the internal nature of the barriers and the possibility of displaying efforts to adopt healthy eating habits. Attributing an outcome to internal causes increases the likelihood of individuals engaging in behavioral change [46,47]. Thus, these girls are expected to make efforts to follow a healthy diet because they believe this goal is within their control. These findings are interesting and stress the distinct factors children select to explain their eating habits (e.g., internal and controllable or external and uncontrollable). In fact, children who select the former reasons are more likely to display an agent role and follow healthy eating habits than their counterparts who select external causes for their eating outcomes [46,48]. Educators could consider developing efforts to help children transform external and uncontrollable barriers into internal and controllable ones.

Current findings should be considered alongside limitations. Results should be interpreted with caution due to the: (1) qualitative nature of the present study; (2) sample selection method, i.e., convenience sample; and (3) specific characteristics of the sample (e.g., geographical area and family income). Another limitation relies on the methodology adopted. Even though the “Top 5 barriers to a healthy diet” is an open-ended question likely to capture the diversity in the reports, most answers were short, consisting of only a few words. Future research may consider including an additional source of data collection to overcome this limitation (e.g., the use of semi-structured interviews or focus groups to deepen our understanding of the phenomenon). Additionally, future research could also consider an open-ended question methodology but without using the scenario/prompt used in the present study.

A healthy diet is a compound that may understate the complexity of barriers that likely prevent children from adopting and maintaining healthy dietary choices. The present study identified a barrier not incorporated in the Six C’s Model [12], i.e., “meal preparation” which was coded in the Clan sphere. Further research should continue exploring the factors and barriers contributing to children’s healthy diets, particularly investigating whether “meal preparation” should be included in the model as a novel factor.

The participants referenced the Child-related barriers most frequently; this finding opens an avenue for setting up healthy diet promotion interventions tailored to help children overcome these barriers. Health practitioners and/or educators may want to consider the complexity of the barriers children perceive when designing and developing future interventions. As current data suggest, interventions could consider targeting child-related factors and barriers, as well as other spheres of context (e.g., parents, peers, and community), to improve intervention efficacy. In particular, self-regulation-based interventions are expected to support children while facing perceived barriers towards healthy eating, both internally and externally. For example, interventions could include hands-on activities that apply self-regulation strategies to meal planning and discuss alternatives to overcome obstacles. To illustrate, an activity could integrate self-regulation strategies in making a colorful and varied meal with playdough (i.e., plan what foods they want to include and, after finishing the dish, evaluate the task, analyzing whether the final product was consistent with the goals set and why) [49,50].

Additionally, advocacy groups are needed to help set environmental and structural changes in the community likely to promote healthy eating for children. Prior research, for example, emphasizes the importance of enacting policies to moderate aggressive marketing that encourages sedentary behavior and unhealthy food and beverage consumption. [51,52]. Based on the present data, it also seems relevant to define public health and policy efforts limiting the opening of food stores and restaurants offering mostly unhealthy foods high in sugar, salt, and fat near schools. In addition, it may be relevant to examine how to reduce the financial barriers to healthy eating, for example, by reducing the price of healthy foods that are, on average, more expensive than unhealthy options [27].

Based on the premise that girls and boys stress distinct barriers to following healthy food paths, educators could consider tailoring interventions to help them overcome these barriers. For example, it is possible that girls will benefit more from interventions focusing on strategies to overcome child-related barriers (e.g., how to reduce excessive media use), while boys will benefit more from interventions focusing on overcoming community-related barriers (e.g., how to choose a healthy food even if the peers choose an unhealthy ones). Moreover, boys may also benefit from interventions aiming to promote healthy diets that display efforts to encourage the child’s agency while helping them transform external and uncontrollable barriers into internal and controllable ones.

## 5. Conclusions

The current study provides a comprehensive ecological perspective on the barriers to a healthy diet as perceived by children. The present results showed that children mostly perceive as barriers to a healthy diet Clan and Community-related factors, such as self-regulation/emotional-regulation, and foods available at home, respectively. Additionally, it seems that girls emphasize more barriers from the Child Sphere, while boys emphasize more barriers from the Community Sphere. This may contribute to a better understanding of healthy eating processes and the development of more effective and tailored interventions and campaigns to promote a healthy diet among children.

## Figures and Tables

**Table 1:** Response frequency by category considering the influence of participants’ attributes of sex (girls vs. boys) and family income (low vs. median/high).

Category	Child Sex		Family Income	
	Girls	Boys	Low	Median/High
	*n* (%)	*n* (%)	*n* (%)	*n* (%)
Child	500 (74)	445 (67)	327 (71)	618 (71)
Clan	53 (8)	31 (5)	19 (4)	65 (7)
Community	55 (8)	112 (17)	69 (15)	98 (11)
Country	51 (8)	57 (8)	36 (8)	72 (8)
Culture	13 (2)	18 (3)	8 (2)	23 (3)

*n* = 1335. Percentages were calculated with respect to the child’s sex and family income attributes.

## Data Availability

Data are available from the first author upon reasonable request.

## References

[1] Chan M. (2020). Ten Years in Public Health, 2007–2017.

[2] Viana V., Guimarães M.J. (2018). Comportamento e hábitos alimentares em crianças e jovens: Uma revisão da literatura. Psicol. Saude Doencas.

[3] Soskolne V., Cohen-Dar M., Obeid S., Cohen N., Rudolf M.C.J. (2018). Risk and Protective Factors for Child Overweight/Obesity Among Low Socio-Economic Populations in Israel: A Cross Sectional Study. Front. Endocrinol..

[4] Swinburn B., Caterson I., Seidell J., James W. (2004). Diet, Nutrition and the Prevention of Excess Weight Gain and Obesity. Public Health Nutr..

[5] World Health Organization Healthy Diet. https://www.who.int/news-room/fact-sheets/detail/healthy-diet.

[6] Kim S.A., Moore L.V., Galuska D., Wright A.P., Harris D., Grummer-Strawn L.M., Merlo C.L., Nihiser A.J., Rhodes D.G. (2014). Vital Signs: Fruit and Vegetable Intake Among Children—United States, 2003–2010. Morb. Mortal. Wkly. Rep..

[7] Pereira B., Silva C., Núñez J.C., Rosário P., Magalhães P. (2021). “More Than Buying Extra Fruits and Veggies, Please Hide the Fats and Sugars”: Children’s Diet Latent Profiles and Family-Related Factors. Nutrients.

[8] Beck A.L., Iturralde E., Haya-Fisher J., Kim S., Keeton V., Fernandez A. (2019). Barriers and Facilitators to Healthy Eating among Low-Income Latino Adolescents. Appetite.

[9] Rastogi S., Mathur P., Khanna A. (2019). Gaps in Nutrition Knowledge and Barriers to Eating Healthy among Low-Income, School-Going Adolescent Girls in Delhi. J. Public Health (Berl.).

[10] Sylvetsky A.C., Visek A.J., Turvey C., Halberg S., Weisenberg J.R., Lora K., Sacheck J. (2020). Parental Concerns about Child and Adolescent Caffeinated Sugar-Sweetened Beverage Intake and Perceived Barriers to Reducing Consumption. Nutrients.

[11] Romero M.Y.M., Jeitner E.C., Francis L.A. (2019). Visualizing Perceived Enablers of and Barriers to Healthy Eating by Youth in Rural El Salvador. J. Nutr. Educ. Behav..

[12] Harrison K., Bost K.K., McBride B.A., Donovan S.M., Grigsby-Toussaint D.S., Kim J., Liechty J.M., Wiley A., Teran-Garcia M., Jacobsohn G.C. (2011). Toward a Developmental Conceptualization of Contributors to Overweight and Obesity in Childhood: The Six-Cs Model. Child Dev. Perspect..

[13] Camacho W.J.M., Díaz J.M.M., Ortiz S.P., Ortiz J.E.P., Morales Camacho M.A., Calderón B.P. (2019). Childhood Obesity: Aetiology, Comorbidities, and Treatment. Diabetes/Metab. Res. Rev..

[14] Swinburn B., Shelly A. (2008). Effects of TV Time and Other Sedentary Pursuits. Int. J. Obes..

[15] Williams S., Greene J. (2018). Childhood Overweight and Obesity: Affecting Factors, Education and Intervention. J. Child. Obes..

[16] Kim H.S., Park J., Ma Y., Im M. (2019). What Are the Barriers at Home and School to Healthy Eating?: Overweight/Obese Child and Parent Perspectives. J. Nurs. Res..

[17] Roberts M., Pettigrew S. (2013). Psychosocial Influences on Children’s Food Consumption: Psychosocial Influences on Children’s Diets. Psychol. Mark..

[18] Borzekowski D.L.G., Pires P.P. (2018). A Six Country Study of Young Children’s Media Exposure, Logo Recognition, and Dietary Preferences. J. Child. Media.

[19] Ozodiegwu I.D., Littleton M.A., Nwabueze C., Famojuro O., Quinn M., Wallace R., Mamudu H.M. (2019). A Qualitative Research Synthesis of Contextual Factors Contributing to Female Overweight and Obesity over the Life Course in Sub-Saharan Africa. PLoS ONE.

[20] de Ridder D., Kroese F., Evers C., Adriaanse M., Gillebaart M. (2017). Healthy Diet: Health Impact, Prevalence, Correlates, and Interventions. Psychol. Health.

[21] Ingoldsby E.M. (2010). Review of Interventions to Improve Family Engagement and Retention in Parent and Child Mental Health Programs. J. Child. Fam. Stud..

[22] Nitsch M., Dimopoulos C.N., Flaschberger E., Saffran K., Kruger J.F., Garlock L., Wilfley D.E., Taylor C.B., Jones M. (2016). A Guided Online and Mobile Self-Help Program for Individuals with Eating Disorders: An Iterative Engagement and Usability Study. J. Med. Internet Res..

[23] Reynolds K., Baranowski T., Bishop D., Farris R., Binkley D., Nicklas T., Elmer P. (1999). Patterns in Child and Adolescent Consumption of Fruit and Vegetables: Effects of Gender and Ethnicity across Four Sites: Journal of the American College of Nutrition: Vol 18, No 3. Am. Coll. Nutr..

[24] Zhang J., Zhai Y., Feng X.Q., Li W.R., Lyu Y.B., Astell-burt T., Zhao P.Y., Shi X.M. (2018). Gender Differences in the Prevalence of Overweight and Obesity, Associated Behaviors, and Weight-Related Perceptions in a National Survey of Primary School Children in China. Biomed. Environ. Sci..

[25] Alkhaldi A.K., Alshiddi H., Aljubair M., Alzahrani S., Alkhaldi A., Al-khalifa K.S., Gaffar B. (2021). Sex Differences in Oral Health and the Consumption of Sugary Diets in a Saudi Arabian Population. Patient Prefer. Adherence.

[26] Vilela S., Muresan I., Correia D., Severo M., Lopes C. (2020). The Role of Socio-Economic Factors in Food Consumption of Portuguese Children and Adolescents: Results from the National Food, Nutrition and Physical Activity Survey 2015–2016. Br. J. Nutr..

[27] Rao M., Afshin A., Singh G., Mozaffarian D. (2013). Do Healthier Foods and Diet Patterns Cost More than Less Healthy Options? A Systematic Review and Meta-Analysis. BMJ Open.

[28] Turrell G., Hewitt B., Patterson C., Oldenburg B. (2003). Measuring Socio-Economic Position in Dietary Research: Is Choice of Socio-Economic Indicator Important?. Public Health Nutr..

[29] Darmon N., Drewnowski A. (2015). Contribution of Food Prices and Diet Cost to Socioeconomic Disparities in Diet Quality and Health: A Systematic Review and Analysis. Nutr. Rev..

[30] Magalhães P., Pereira B., Holmes C., Silva C., Rosário P., Wilson L. (2020). Using Google Classroom to Foster User Engagement in a Hybrid Healthy Eating Promotion Intervention Among Elementary School Students: Lessons Learned. Healthy lifestyles and Healthy Eating.

[31] Milheiro C. Ação Social Escolar: Tudo o que Precisa de Saber Para 2021–2022. https://www.e-konomista.pt/acao-social-escolar/.

[32] Campbell J.M., Morton J.F., Roulston K., Barger B.D. (2011). A Descriptive Analysis of Middle School Students’ Conceptions of Autism. J. Dev. Phys. Disabil..

[33] Creswell J. (2012). Educational Research: Planning, Conducting, and Evaluating Quantitative and Qualitative Research.

[34] Bardin L. (1977). The Content Analysis.

[35] Pereira B., Rosário P., Núñez J.C., Rosendo D., Roces C., Magalhães P. (2021). Food Availability, Motivational-Related Factors, and Food Consumption: A Path Model Study with Children. Int. J. Environ. Res. Public Health.

[36] Watts A.W., Lovato C.Y., Barr S.I., Hanning R.M., Mâsse L.C. (2015). Experiences of Overweight/Obese Adolescents in Navigating Their Home Food Environment. Public Health Nutr..

[37] Scaglioni S., De Cosmi V., Ciappolino V., Parazzini F., Brambilla P., Agostoni C. (2018). Factors Influencing Children’s Eating Behaviours. Nutrients.

[38] Sharif Ishak S.I.Z., Chin Y.S., Mohd Taib M.N., Mohd Shariff Z. (2020). Malaysian Adolescents’ Perceptions of Healthy Eating: A Qualitative Study. Public Health Nutr..

[39] Vaughn A.E., Martin C.L., Ward D.S. (2018). What Matters Most - What Parents Model or What Parents Eat?. Appetite.

[40] Sugimura K., Hihara S., Hatano K. (2020). Emotional Separation, Parental Trust, and Psychosocial Adjustment in Preadolescence and Early Adolescence. J. Adolesc..

[41] Pereira B., Rosário P., Silva C., Figueiredo G., Núñez J.C., Magalhães P. (2019). The Mediator and/or Moderator Role of Complexity of Knowledge about Healthy Eating and Self-Regulated Behavior on the Relation between Family’s Income and Children’s Obesity. IJERPH.

[42] Magalhães P., Pereira B., Dembo R., Silva C., Machado P., Rosário P. (2020). The Overlooked Role of Motivation Related Variables on Children’s Healthy Eating. Healthy Lifestyles and Healthy Eating.

[43] Musaiger A.O., Al-Mannai M., Tayyem R., Al-Lalla O., Ali E.Y.A., Kalam F., Benhamed M.M., Saghir S., Halahleh I., Djoudi Z. (2013). Perceived Barriers to Healthy Eating and Physical Activity among Adolescents in Seven Arab Countries: A Cross-Cultural Study. Sci. World J..

[44] de Vet E., de Wit J.B.F., Luszczynska A., Stok F.M., Gaspar T., Pratt M., Wardle J., De Ridder D.T.D. (2013). Access to Excess: How Do Adolescents Deal with Unhealthy Foods in Their Environment?. Eur. J. Public Health.

[45] Amiri P., Ghofranipour F., Ahmadi F., Hosseinpanah F., Montazeri A., Jalali-Farahani S., Rastegarpour A. (2011). Barriers to a Healthy Lifestyle among Obese Adolescents: A Qualitative Study from Iran. Int. J. Public Health.

[46] Almeida L., Miranda L., Guisande M. (2008). Atribuições Causais Para o Sucesso e Fracasso Escolares Atribuições Causais Para o Sucesso e Fracasso Escolares. Estud. Psicol. Camp..

[47] Graham S. (2020). An Attributional Theory of Motivation. Contemp. Educ. Psychol..

[48] Weiner B. (1985). An Attributional Theory of Achievement Motivation and Emotion. Psychol. Rev..

[49] Pereira B., Magalhães P., Pereira R. (2018). Building Knowledge of Healthy Eating in Hospitalized Youth: A Self-Regulated Campaign. Psicothema.

[50] Magalhães P., Silva C., Pereira B., Figueiredo G., Guimarães A., Pereira A., Rosário P. (2020). An Online-Based Intervention to Promote Healthy Eating through Self-Regulation among Children: Study Protocol for a Randomized Controlled Trial. Trials.

[51] Lobstein T., Jackson-Leach R., Moodie M., Hall K., Gortmaker S., Swinburn B., James W., Wang Y., McPherson K. (2015). Child and Adolescent Obesity: Part of a Bigger Picture. Lancet.

[52] Swinburn B.A., Sacks G., Hall K.D., McPherson K., Finegood D.T., Moodie M.L., Gortmaker S.L. (2011). The Global Obesity Pandemic: Shaped by Global Drivers and Local Environments. Lancet.

